# Identification of the central symptoms of neuropsychiatric symptoms among older adults with mild cognitive impairment: a network analysis

**DOI:** 10.3389/fpsyt.2026.1820870

**Published:** 2026-04-29

**Authors:** Kexin Huang, Wei Liu, Zhenlim Wong, Yuhang Pu, Qinghuan Kong, Rui Han, Rendong He, Yong Jia, Li Chen

**Affiliations:** 1 School of Nursing, Jilin University, Changchun, China; 2 School of Nursing, Beihua University, Jilin, China; 3 The Sidney Kimmel Comprehensive Cancer Center, Johns Hopkins University School of Medicine, Baltimore, MD, United States

**Keywords:** mild cognitive impairment, network analysis, neuropsychiatric symptoms, older adults, symptom network

## Abstract

**Background:**

Neuropsychiatric symptoms (NPSs) are prevalent among older adults with mild cognitive impairment (MCI) and are associated with accelerated dementia progression and increased caregiving burden. However, existing studies have mostly focused on total scores or symptom severity, with no study to date using network analysis to identify central symptoms within NPS in older adults with MCI.

**Design:**

A cross-sectional study was conducted from September 2025 to January 2026 in China.

**Settings and participants:**

Older adults with MCI in China were recruited.

**Methods:**

NPSs were evaluated using the Neuropsychiatric Inventory Questionnaire (NPI-Q). Symptom networks were estimated in *R* using the q-graph and IsingFit packages. Centrality indices including strength, betweenness, closeness, and expected influence, were computed to identify the central symptoms in the NPS network among older adults with MCI.

**Results:**

A total of 617 older adults with MCI were recruited, of whom 559 were included in the final analysis. Among these participants, 385 (68.9%) were identified as having at least one NPS. The sample comprised 261 females (46.7%), with a median age of 70.0 years. Centrality analyses indicates that disinhibition, irritability/lability, and agitation/aggression had the highest strength and expected influence values, suggesting that these symptoms were the most central symptoms within the NPS network.

**Conclusion:**

This study primarily provided network analysis to construct a symptom network of NPSs among older adults with MCI. The findings revealed that disinhibition, irritability/lability, and agitation/aggression occupied relatively central positions in the network. Prioritizing the assessment and intervention of these symptoms may help inform more focused and efficient approaches to NPSs management in MCI.

**Patient or public contribution:**

No patient or public contribution was involved in this study.

## 1 Introduction

With the rapid ageing of the global population, dementia is increasingly becoming a major public health priority, and now one of the leading causes of disability in the older adults and a substantial driver of increasing health-care, and societal costs and burden worldwide (1, 2). The number of people aged 60 years and older is projected to reach approximately 2.1 billion by 2050 (3). As such, China will become the country with the largest number of older adults with dementia, accounting for nearly one-quarter of the global dementia population, with this number projected to exceed 30 million by 2050 (1, 2, 4). Therefore, an effective early identification of individuals at high risk of dementia and the implementation of targeted interventions have become central to improving dementia-related care outcomes.

Neuropsychiatric symptoms (NPSs) are the core clinical feature of prodromal and clinical dementia, and they are closely associated with a wide range of adverse clinical outcomes, as well as substantially increasing the complexity of clinical management of the disease. NPS refer to behavioral, affective, and personality changes attributable to underlying neurodegenerative processes, commonly including apathy, depression, aggression, anxiety, and sleep disturbance, among others (5). NPSs were often assessed using the NPI-Q, which captures the presence and severity of 12 symptom domains over the period of a month (6). Studies indicates that up to 97% of individuals with dementia experience at least one NPS (7), which are linked to increased caregiving burden (8), greater functional impairment (9), higher rates of institutionalization (10), reduced quality of life (11), and a faster progression to later stages of dementia and ultimately death (12).

NPSs were previously considered to be observed primarily in the middle or latter stages of dementia, however, there are accumulating evidence now indicating that NPSs can also be observed during the early phases of dementia and even in some older adults with mild cognitive impairment (MCI) (13, 14). Furthermore, the presence of NPSs in these individuals significantly increases the risk of progression from MCI to dementia (15). As a critical transitional stage between normal cognition and dementia, MCI is characterized not only by cognitive decline but also by a substantial neuropsychiatric burden, with NPS prevalence estimates ranging from 35% to 85%, and more than half of individuals with MCI exhibiting at least one NPS (16). Moreover, NPSs do not occur in isolation but demonstrate non-random clustering and dynamic interrelationships, whereby one symptom may exacerbate or transition into another symptom, forming an interconnected symptom system (16, 17). Consequently, focusing solely on the prevalence or overall severity of these symptoms provides limited guidance for prioritizing clinical interventions. Evidence from Alzheimer’s disease (AD) populations shows that NPSs tend to co-occur as symptom clusters or subsyndromes (18). In individuals with MCI, affective and psychotic symptom domains have been shown to predict an increased risk of clinical dementia, with synergistic effects observed when these domains coexist (19). Moreover, the co-occurrence of multiple NPSs further elevates the risk of progression to AD, with apathy being shown as a particularly significant and independent predictor of conversion and adverse outcomes, whereas depression appears to be a weaker factor towards risk of disease progression (20, 21).

Symptom network models provide an effective methodological framework to understand the interdependence of NPSs and identify crucial symptoms within complex symptom systems (22). In this framework, individual symptoms are conceptualized as nodes, and statistical associations between symptoms are represented as edges, together forming an interconnected network of symptom interactions (23). A “central symptom” refers to a symptom that occupies a relatively influential position within the network, typically indicated by centrality indices such as strength, closeness, or betweenness (24). Such symptoms are more strongly or more broadly connected with other symptoms in the network and may serve as strategic targets for precision clinical diagnosis and intervention (24). The presence or exacerbation of central symptoms is more likely to propagate effects across the network, offering a novel evaluative perspective beyond symptom prevalence and severity for identifying sentinel symptoms with prognostic significance.

To date, symptom network analysis has been successfully applied in studies of depression and anxiety as well as schizophrenia, supporting its validity and clinical relevance across various psychiatric disorders (25, 26).

More recently, a limited number of studies have applied network analysis to explore the structural characteristics of NPSs. However, they are still substantial limitations in them, including the clarification between symptom interrelationships and identification of central symptoms within individuals with MCI. Existing network-based studies have predominantly focused on patients with AD, populations at high risk of dementia, individuals with mild behavioral impairment, or mixed samples combining MCI and AD. This results in the NPS network structure in MCI-specific older adult populations largely unexplored. Goodwin et al. reported that disinhibition and agitation/aggression were the most central symptoms in a mixed MCI-AD sample, whereas depression did not emerge as a central node (27). Conversely, Wei et al. identified anhedonia as the core symptom in individuals with mild behavioral impairment, with distinct cross-domain bridge symptoms, highlighting heterogeneity across clinical populations (17). Other studies have also showed that under different depressive status, there will be differences in the NPSs networks and central symptoms further demonstrating the population-specific nature of NPS network structures (28, 29).

As such, this study aimed to construct an item-level NPS network based on the NPI-Q in older adults with MCI. Participants with MCI were first identified and subsequently categorized into the MCI-NPS+ and MCI-NPS− groups according to the presence or absence of NPS to characterize subgroup differences within the overall MCI sample. The primary analysis focused on participants with at least one NPS (MCI-NPS+ group). Specifically, the objectives were to: (1) explore the interrelationships among NPS; and (2) identify potentially central symptoms within the network. Elucidating the NPS network structure at the MCI stage may provide insights into patterns of symptom co-occurrence and inform future research on symptom monitoring and targeted intervention strategies.

## 2 Methods

### 2.1 Study design and settings

This study is a cross-sectional, observational investigation conducted within a network analysis framework, aiming to examine the structure and interrelationships of neuropsychiatric symptoms among older adults with MCI in both community and residential care settings. The study was reported in accordance with the Strengthening the Reporting of Observational Studies in Epidemiology (STROBE) guidelines. Convenience sampling was used, and data were collected between September 2025 and January 2026 in Changchun, Jilin Province, China. Changchun is located in northeastern China and has a total population of approximately 9.10 million, of which about 1.89 million (20.85%) are aged 60 years or older.

To enhance sample representativeness, a dual-mode recruitment and data collection strategy was adopted, combining face-to-face interviews with online questionnaires. Specifically, trained investigators conducted face-to-face surveys in three central urban districts of Changchun (Chaoyang, Nanguan, and Kuancheng) and in four residential care facilities, while online questionnaires were distributed through the WenJuanXing electronic survey platform (https://www.wjx.cn/) to reach older adults from both urban and rural communities. The online stage was used solely to collect sociodemographic and clinical characteristics and to identify potentially eligible older adults. After this preliminary screening, all eligible participants were invited to complete face-to-face assessments for standardized evaluation of MCI and NPS. In these face-to-face assessments, the Montreal Cognitive Assessment (MoCA) was administered by trained researchers, the Activities of Daily Living (ADL) scale was assessed through structured interviews based on participant self-report or caregiver report, and the NPI-Q was completed by caregivers. Online surveys incorporated same-IP/device restrictions, a minimum completion-time threshold, and attention-check items, while face-to-face interviews included on-site verification and post-collection data audits to ensure data quality and comparability. Participants with incomplete key assessment data (e.g., missing NPI-Q data) were excluded from the final analysis. A complete-case analysis was performed, and no imputation for missing data was conducted. The study design and procedures adhered to the principles of the Declaration of Helsinki. Ethical approval was obtained from the ethics committee of the authors’ university (Approval No. 2025091701). Written informed consent was obtained from all participants prior to participation.

### 2.2 Participants

Inclusion criteria for older adults with MCI were as follows: (1) Community-dwelling or residential care settings adults aged ≥60 years. (2) MCI was operationally defined according to the following criteria: ① Subjective cognitive decline reported by the participant and a primary informant; ② Objective cognitive impairment on screening, assessed using the MoCA, defined as a total score <26, with an additional point added for individuals with ≤12 years of education in accordance with standard guidelines; ③ Preserved basic functional independence, assessed using the ADL scale, with scores <26 indicating no significant functional impairment; ④ Absence of a prior clinical diagnosis of dementia. (3) Clear consciousness and adequate communication ability. (4) Provision of written informed consent and voluntary participation. Exclusion criteria included: (1) A diagnosis of severe psychiatric disorders or cognitive impairment. (2) Severe hearing or visual impairment.

After enrollment, participants were further categorized according to the presence or absence of NPS, as assessed using the Neuropsychiatric Inventory Questionnaire (NPI-Q). Participants with a score >0 in at least one symptom domain (frequency ≥1 and severity ≥1) were classified as the MCI-NPS+ group, whereas those with no reported NPS were classified as the MCI-NPS− group. Because the primary aim of the network analysis was to examine the interrelationships among NPS in older adults with MCI and identify central symptoms, only participants in the MCI-NPS+ group were included in the subsequent symptom network analysis.

Sample size estimation for network analysis followed the recommendations of Constantin (30) and was determined using the formula *n* = *N* × (*N* − 1)/2, where *N* represents the number of nodes. In this study, the NPI-Q comprises 12 items, yielding 66 pairwise parameters. Assuming 3–5 observations per parameter, the minimum required sample size was estimated to range from 198 to 330 participants.

### 2.3 Measures

#### 2.3.1 Sociodemographic and clinical characteristics of the participants

Participants’ sociodemographic and clinical characteristics were collected using self-administered questionnaires, with assistance from or completion by primary caregivers when necessary (**Appendix S1**). These data were obtained through a combination of online and face-to-face approaches. Sociodemographic variables included age, gender, body mass index (BMI), ethnicity, religious belief, education level, location, marital status, employment status, living conditions, and monthly income. Health-related behaviors included smoking, drinking alcohol, self-rated recent health status, and social support. Clinical information comprised medical history of 22 common chronic conditions and a parental history of dementia.

#### 2.3.2 Montreal cognitive assessment

Cognitive function was assessed using the MoCA (31), which can evaluate multiple cognitive domains, including visuospatial/executive function, naming, memory, attention, language fluency, abstraction, delayed recall, and orientation. Total scores range from 0 to 30, with higher scores indicating better cognitive performance. Consistent with standard criteria, scores >26 were considered cognitively normal, whereas scores <26 indicated cognitive impairment; a one-point correction was applied for individuals with fewer than 12 years of education. The Chinese version of the MoCA has demonstrated good criterion validity and satisfactory internal consistency (Cronbach’s α = 0.807) (32). In the present study, the MoCA was used as the objective cognitive screening tool for MCI, and all assessments were administered face-to-face by trained researchers following standardized procedures.

#### 2.3.3 Activities of daily living

ADL were assessed using a 20-item ADL scale adapted and Chinese-translated by Zhang Mingyuan, based on the Lawton and Brody ADL scale. The scale evaluates functional independence across 20 items, each rated on a 4-point Likert scale ranging from 1 (complete independence) to 4 (complete dependence). Total scores range from 20 to 80, with higher scores indicating greater functional impairment, and scores >26 were considered indicative of impaired daily functioning. The scale has demonstrated excellent internal consistency when applied in individuals with MCI, with a Cronbach’s α of 0.92, supporting its reliability in this population (33). In the present study, ADL was assessed face-to-face through structured interviews conducted by trained researchers, based on participant self-report or caregiver report.

#### 2.3.4 Neuropsychiatric inventory questionnaire

NPSs were assessed using the NPI-Q, a brief informant-based instrument developed by Kaufer et al. (6) as a shortened version of the NPI (6). The Chinese version was translated and validated by Ma et al. (34) (34). The NPI-Q assesses 12 common neuropsychiatric symptom domains: delusions, hallucinations, agitation/aggression, depression/dysphoria, anxiety, elation/euphoria, apathy/indifference, disinhibition, irritability/lability, aberrant motor activity, night behavioral disturbances, appetite/eating abnormalities. In this study, the NPI-Q was completed by caregivers, who reported the presence of each symptom during the past month and rated its severity on a 3-point scale (1 = mild, 2 = moderate, 3 = severe), reflecting the degree to which the symptom affected the patient. Symptom domain scores corresponded to severity ratings, and the total NPI-Q score was calculated as the sum of scores across all 12 domains. The number of NPS was defined as the count of symptom domains with a severity score greater than zero. The NPI-Q has demonstrated good internal consistency in older adults with MCI in Chinese nursing-home settings, with a reported Cronbach’s α of 0.841 (35), supporting its reliability in this population.

### 2.4 Statistical analysis

#### 2.4.1 Descriptive analysis

Prior to formal statistical testing, all variables were examined for distributional properties and outliers. Normality of continuous variables was assessed using the Shapiro-Wilk test, and homogeneity of variances was evaluated using Levene’s test. Continuous variables that met assumptions of normality and homoscedasticity are presented as mean ± standard deviation (*SD*) and were compared between participants with NPSs (MCI-NPS+) and those without NPSs (MCI-NPS−) using independent-samples *t* tests. Continuous variables that did not meet normality assumptions are reported as median (interquartile range [*IQR*]) and were compared using the Mann-Whitney U test. Categorical variables are expressed as frequencies (percentages) and were compared between groups using the chi-square (*χ*²) test. Comparisons between the MCI-NPS− and MCI-NPS+ groups were conducted to characterize subgroup differences within the overall MCI sample and were not intended to explain the symptom network.

#### 2.4.2 Constructions of the symptom network

To examine the interrelationships among NPSs in older adults with MCI, we constructed a symptom network based 12 NPSs on using the Ising model, which is specifically suited for binary data (36, 37). Each item of the NPI-Q was dichotomized (0 = absence of the symptom; 1 = presence of the symptom) to fit the model requirements.

Prior to network estimation, topological overlap was assessed to ensure that the included symptoms represented unique constructs rather than redundant indicators. The goldbricker function from the R package networktools was applied to compare correlation patterns across items and detect topological redundancy. A similarity proportion threshold of 0.25 and a statistical significance level of *p* < 0.01 were used to determine whether two items should be retained as independent nodes (38).

The Ising model was estimated using the extended Least Absolute Shrinkage and Selection Operator (eLasso) procedure implemented in the IsingFit package in R. The eLasso approach fits a series of L1-regularized logistic regression models for each symptom node and applies the extended Bayesian information criterion (EBIC) for model selection, yielding a sparse and interpretable network structure that retains relatively robust conditional dependencies between symptoms (37, 39). The EBIC hyperparameter was set to *γ* = 0.25. In the resulting network, each NPS represents a node, and edges reflect pairwise conditional dependencies between symptoms after controlling for all other symptoms (40). Green and red edges denote positive and negative associations, respectively, while edge thickness indicates the magnitude of the estimated relationship (40).

To visualize the network structure, we used the qgraph package in R with a Fruchterman-Reingold spring layout, such that more strongly connected symptoms were positioned closer together, facilitating interpretation of the multidimensional structure of NPS.

#### 2.4.3 Node centrality and expected influence

To identify the most influential NPS within the network, we calculated three commonly used node centrality indices: strength, betweenness, and closeness (41). Strength was defined as the sum of the absolute edge weights connected to each node and reflects the extent to which a symptom co-occurs or interacts with other symptoms. Betweenness quantified how frequently a node lies on the shortest paths between all other pairs of nodes, indicating its role as a bridge linking different symptom clusters. Closeness represented the inverse of the average shortest path length from a node to all other nodes, reflecting how efficiently a symptom can influence or reach other symptoms across the network.

Because the symptom network contained both positive and negative associations, we additionally computed expected influence (*EI*) to better capture the overall influence of each node (42). Specifically, *EI* was defined as the sum of all edge weights directly connected to a given node, with positive and negative values retained rather than transformed into absolute values. This measure reflects the overall influence of a symptom on its neighboring symptoms, taking into account both activating and inhibiting associations.

Symptoms with higher centrality indices and higher *EI* values were considered more influential and were interpreted as potential core symptoms within the NPS network of older adults with MCI.

#### 2.4.4 Network accuracy and stability

To systematically evaluate the reliability of the constructed symptom network, we conducted network accuracy and stability analyses using the bootnet package in R (36). First, the precision of the estimated edge weights was assessed by performing 1,000 nonparametric bootstrap samples to obtain 95% confidence intervals (*CI*s) for each edge. Narrower *CI*s indicated greater precision and stability in the edge-weight estimates.

Subsequently, we applied the case-dropping subset bootstrap procedure (1,000 samples) to assess the stability of the EI of nodes. The primary index of stability was the correlation stability coefficient (CS-C). CS-C values should not fall below 0.25, while values above 0.50 indicate desirable stability and allow for confident interpretation of the results (43).

## 3 Results

### 3.1 Participants

A total of 617 older adults with MCI were initially recruited for this study. Of these, 58 participants were excluded, including 31 who declined to participate and 27 with incomplete NPI-Q data. Consequently, 559 participants were included in the final analysis. Among them, 385 participants (68.9%) reported at least one NPS and were classified into the MCI-NPS+ group, while 174 participants (31.1%) reported no NPS and were classified into the MCI-NPS− group. Given that the primary aim of this study was to examine the interrelationships among NPS and identify central symptoms within the symptom network, only participants in the MCI-NPS+ group (*n* = 385) were included in the subsequent symptom network analysis. The flowchart of the recruitment process is shown in **Appendix S2**.

### 3.2 Sociodemographic and clinical characteristics of the sample

Among the 559 older adults with MCI, 261 (46.7%) were female. The median age was 70.0 years, and the median body mass index (BMI) was 22.21 kg/m². Most participants were of Han ethnicity (91.1%), and 10.4% reported having a religious belief. More than half of the sample had attained a junior high school education or higher. With respect to location, 51.2% lived in urban areas and 48.8% in non-urban areas. Most participants were married (75.9%). The predominant employment status was retired (55.7%), followed by unemployed (31.8%), and only 5.0% were currently employed. In terms of living conditions, 75.8% lived with spouse. Regarding socioeconomic status, the majority reported a monthly income of ≤¥2,000 (37.4%) or ¥2,001-¥5,000 (35.2%). In terms of health behaviors, 42.8% reported a history of smoking and 43.5% reported alcohol consumption. Self-rated health status was generally favorable, with 52.2% rating their health as “healthy”, 35.6% as “not healthy, not unhealthy” and 12.2% as “unhealthy”. Most participants reported receiving social support (74.8%). Chronic disease burden was prevalent, 44.9% had two or more chronic conditions, 36.0% had one chronic condition, and 19.1% reported none. A parental history of dementia was reported by 3.9% of participants. Detailed participant characteristics are presented in **Table 1**.

**Table 1:** Sociodemographic characteristics for the participants (*N* = 559).

Characteristics	Category	NPS classification				
		Total sample (*N* = 559)	MCI-NPS− (*n* = 174)	MCI-NPS+ (*n* = 385)	*χ*^2^/*U*	*p*-value
Age (years, median, q1, q3)		70.00 (64.00, 76.00)	71.00 (65.00, 77.00)	69.00 (63.00, 74.00)	-3.117^a^	0.002
	60–69 years (*n*, %)	264 (47.2)	94 (54.0)	170 (44.2)		
	70–79 years (*n*, %)	207 (37.0)	60 (34.5)	147 (38.2)		
	≥ 80 years (*n*, %)	88 (15.8)	20 (11.5)	68 (17.6)		
Gender (*n*, %)					0.019^b^	0.890
	Male	298 (53.3)	92 (52.3)	206 (53.5)		
	Female	261 (46.7)	82 (47.7)	179 (46.5)		
BMI (median, q1, q3)		22.21 (20.32, 26.65)	22.06 (20.06, 24.06)	23.36 (20.77, 25.73)	-3.954 ^a^	< 0.001
Ethnicity (*n*, %)					1.301 ^b^	0.254
	Han Chinese	509 (91.1)	162 (93.1)	347 (90.1)		
	Ethnic minority	50 (8.9)	12 (6.9)	38 (9.9)		
Religious belief (*n*, %)					55.844 ^b^	< 0.001
	Yes	58 (10.4)	43 (24.7)	15 (3.9)		
	No	501 (89.6)	131 (75.3)	370 (96.1)		
Education level (*n*, %)					8.638 ^b^	0.071
	Illiterate	109 (19.5)	37 (21.3)	72 (18.7)		
	Primary elementary	135 (24.2)	42 (24.1)	93 (24.2)		
	Junior high school	198 (35.4)	71 (40.8)	127 (33.0)		
	Senior high school	65 (11.6)	13 (7.5)	52 (13.5)		
	Graduate or above	52 (9.3)	11 (6.3)	41 (10.6)		
Location (*n*, %)					5.664 ^b^	0.017
	Urban	286 (51.2)	76 (43.7)	210 (54.5)		
	Non-urban	273 (48.8)	98 (56.3)	175 (45.4)		
Marital status (*n*, %)					0.721 ^b^	0.396
	Unmarried	135 (24.1)	46 (26.4)	89 (23.1)		
	Married	424 (75.9)	128 (73.6)	296 (76.9)		
Employment status (*n*, %)					45.851 ^b^	< 0.001
	Employed	28 (5.0)	12 (6.9)	16 (4.1)		
	Retired	311 (55.7)	64 (36.8)	247 (64.2)		
	Unemployed	178 (31.8)	71 (40.8)	107 (27.8)		
	Others	42 (7.5)	27 (15.5)	15 (3.9)		
Living conditions (*n*, %)					14.414 ^b^	0.006
	Live alone	35 (6.3)	4 (2.3)	31 (8.1)		
	Live with parents	13 (2.3)	4 (2.3)	9 (2.3)		
	Live with children	56 (10.1)	26 (14.9)	30 (7.8)		
	Live with spouse	424 (75.8)	127 (73.0)	297 (77.1)		
	Live with other friends	31 (5.5)	13 (7.5)	18 (4.7)		
Monthly income (*n*, %)					26.032 ^b^	< 0.001
	¥2000 or less	209 (37.4)	83 (47.7)	126 (32.7)		
	¥2001-¥5000	197 (35.2)	66 (37.9)	131 (34.0)		
	¥5001-¥10000	116 (20.8)	23 (13.3)	93 (24.2)		
	¥10001 or more	37 (6.6)	2 (1.1)	35 (9.1)		
Smoking (*n*, %)					5.469 ^b^	0.019
	Yes	239 (42.8)	62 (35.6)	177 (46.0)		
	No	320 (57.2)	112 (64.4)	208 (54.0)		
Drinking alcohol (*n*, %)					5.424 ^b^	0.020
	Yes	243 (43.5)	63 (36.2)	180 (46.8)		
	No	316 (56.5)	111 (63.8)	205 (53.2)		
Self-reported health status (*n*, %)					27.693 ^b^	< 0.001
	Healthy	292 (52.2)	49 (28.2)	223 (57.9)		
	Not healthy, not unhealthy	199 (35.6)	67 (38.5)	132 (34.3)		
	Unhealthy	68 (12.2)	38 (21.8)	30 (7.8)		
Social support (*n*, %)					0.748 ^b^	0.387
	Yes	418 (74.8)	126 (72.4)	292 (75.8)		
	No	141 (25.2)	48 (27.6)	93 (24.2)		
Number of chronic diseases (*n*, %)					5.789 ^b^	0.055
	Zero	107 (19.1)	34 (19.6)	73 (19.0)		
	One	201 (36.0)	74 (42.5)	127 (33.0)		
	Two or more	251 (44.9)	66 (37.9)	185 (48.0)		
Parental history of dementia (*n*, %)					3.191 ^b^	0.074
	Yes	22 (3.9)	3 (1.7)	19 (4.9)		
	No	537 (96.1)	171 (98.3)	366 (95.1)		

a ***χ*^2^**.

b U.

November 13th, 2025: 1.00 RMB = 0.14$; 1.00 RMB = 0.11£.

q1, 25th percentile; q3, 75th percentile; MCI, Mild Cognitive Impairment; NPS, Neuropsychiatric Symptoms; MCI-NPS−, MCI without neuropsychiatric symptoms; MCI-NPS+, MCI with at least one neuropsychiatric symptom.

To characterize subgroup differences within the overall MCI sample, demographic and clinical characteristics were compared between the MCI-NPS− and MCI-NPS+ groups. Significant differences were observed in age, BMI, religious belief, location, employment status, living conditions, monthly income, smoking, drinking alcohol, and self-reported health status (all *p* < 0.05; **Table 1**).

### 3.3 Symptom prevalence of NPS among older adults with MCI (*n* = 385)

**Table 2** presents the number and proportion of NPS assessed using individual items of the NPI-Q. Among the 12 NPI domains, irritability/lability was the most prevalent symptom (*n* = 269, 69.9%), followed by anxiety (*n* = 182, 47.3%) and apathy/indifference (*n* = 172, 44.7%).

**Table 2:** Symptom prevalence of NPS among older adults with MCI (*N* = 385).

Neuropsychiatric inventory	*n* (%)
Delusions	23 (6.0)
Hallucinations	27 (7.0)
Agitation/aggression	93 (24.2)
Depression/Dysphoria	122 (31.7)
Anxiety	182 (47.3)
Elation/euphoria	25 (6.5)
Apathy/indifference	172 (44.7)
Disinhibition	55 (14.3)
Irritability/lability	269 (69.9)
Aberrant motor activity	25 (6.5)
Night behavioral disturbances	161 (41.8)
Appetite/eating abnormalities	60 (15.6)

### 3.4 Network structure among the NPI items and centrality indices (*n* = 385)

**Figure 1:**
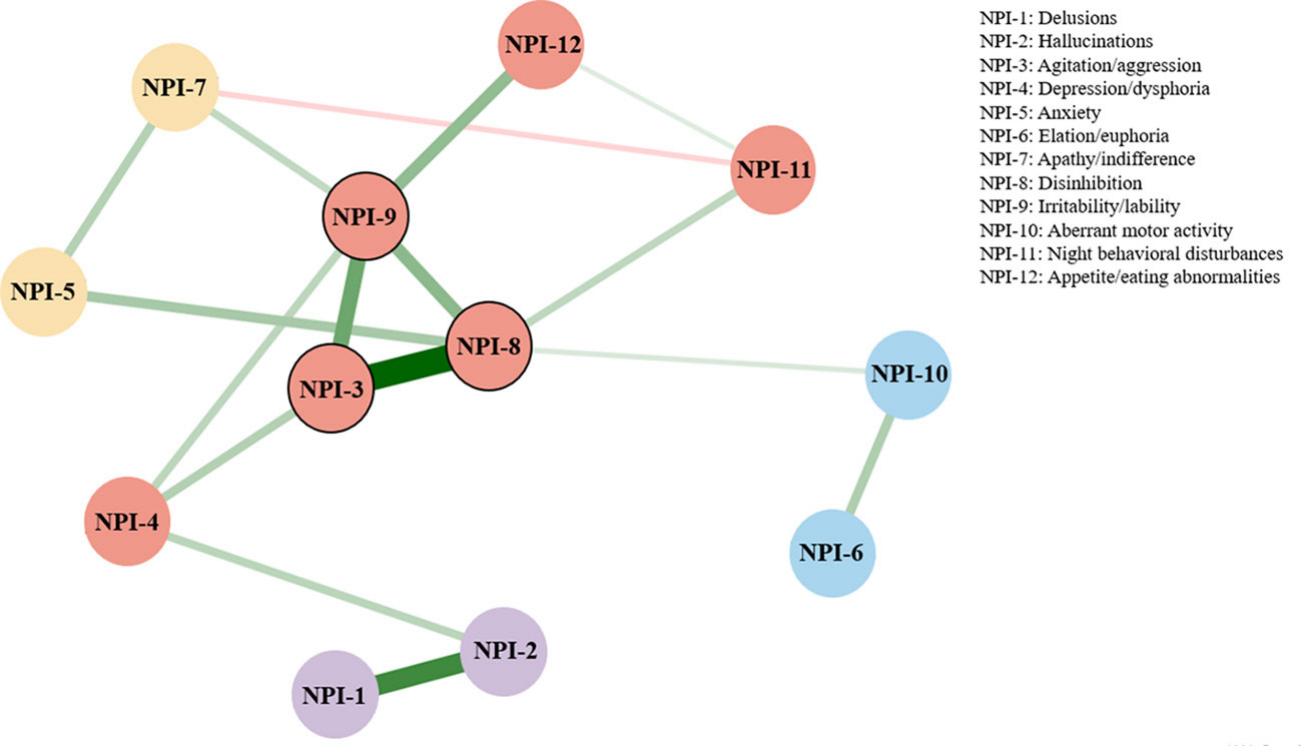
The symptom network of NPS among older adults with MCI. Note. Edges represent regularized partial correlation coefficients between symptoms, with green edges indicating positive associations and red edges indicating negative associations. Edge thickness and color intensity reflect the absolute magnitude of the associations. Node colors were manually assigned to illustrate clusters derived from the network structure. Nodes with highest strength centrality and expected influence are outlined in black.

**Figure 2:**
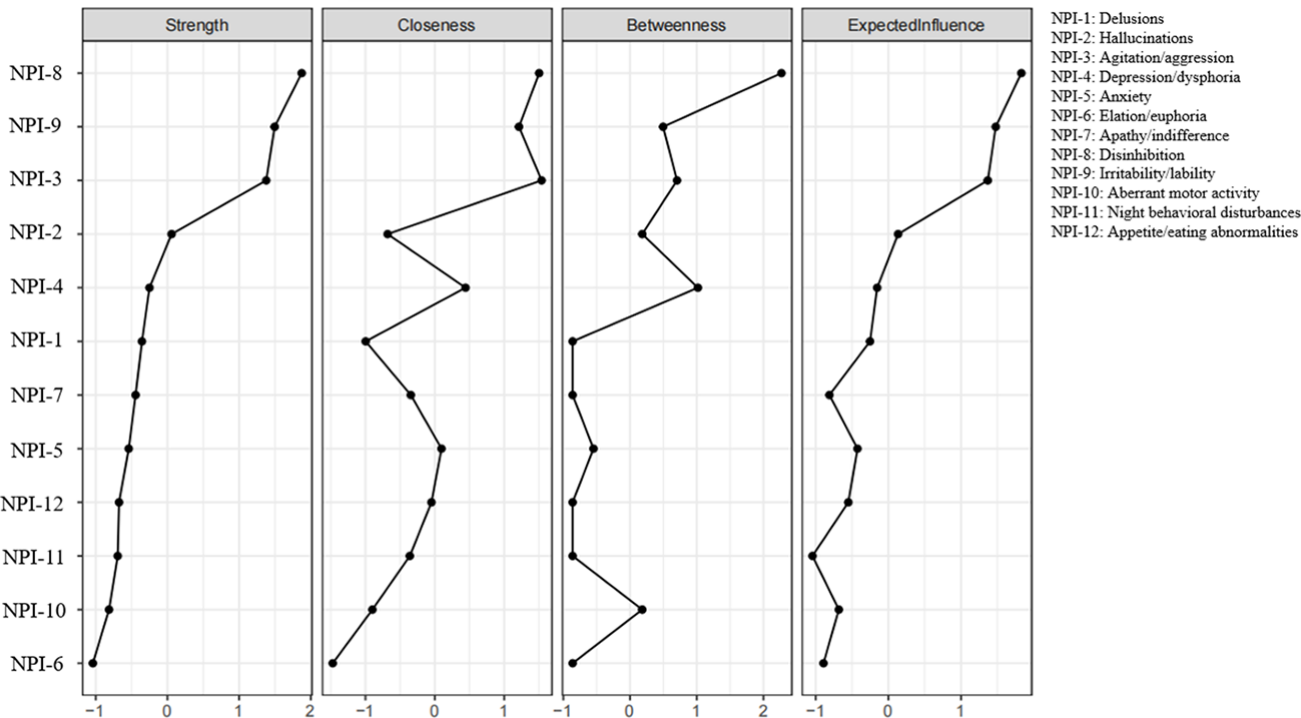
The centrality indices of 12 symptoms.

The Ising model was applied to construct the NPS symptom network among older adults with MCI. The estimated symptom network is presented in **Figure 1**, and centrality indices are shown in **Figure 2**. Strength and EI centrality indicated that disinhibition (*rs* = 6.823, *EI* = 6.823), irritability/lability (*rs* = 6.065, *EI* = 6.065), and agitation/aggression (*rs* = 5.829, *EI* = 5.829) were the most influential symptoms in the network, suggesting that they were most strongly connected to other symptoms. In terms of closeness centrality, agitation/aggression (*rc* = 0.052), disinhibition (*rc* = 0.052), and irritability/lability (*rc* = 0.049) ranked highest. Regarding betweenness centrality, disinhibition (*rb* = 30), depression/dysphoria (*rb* = 18), and agitation/aggression (*rb* = 15) showed the highest values. No symptoms were identified for removal during network estimation. Full centrality metrics for all NPS items are provided in **Supplementary Appendix S3t**.

### 3.5 Stability, accuracy and difference tests of symptom networks (*n* = 385)

**Figure 3:**
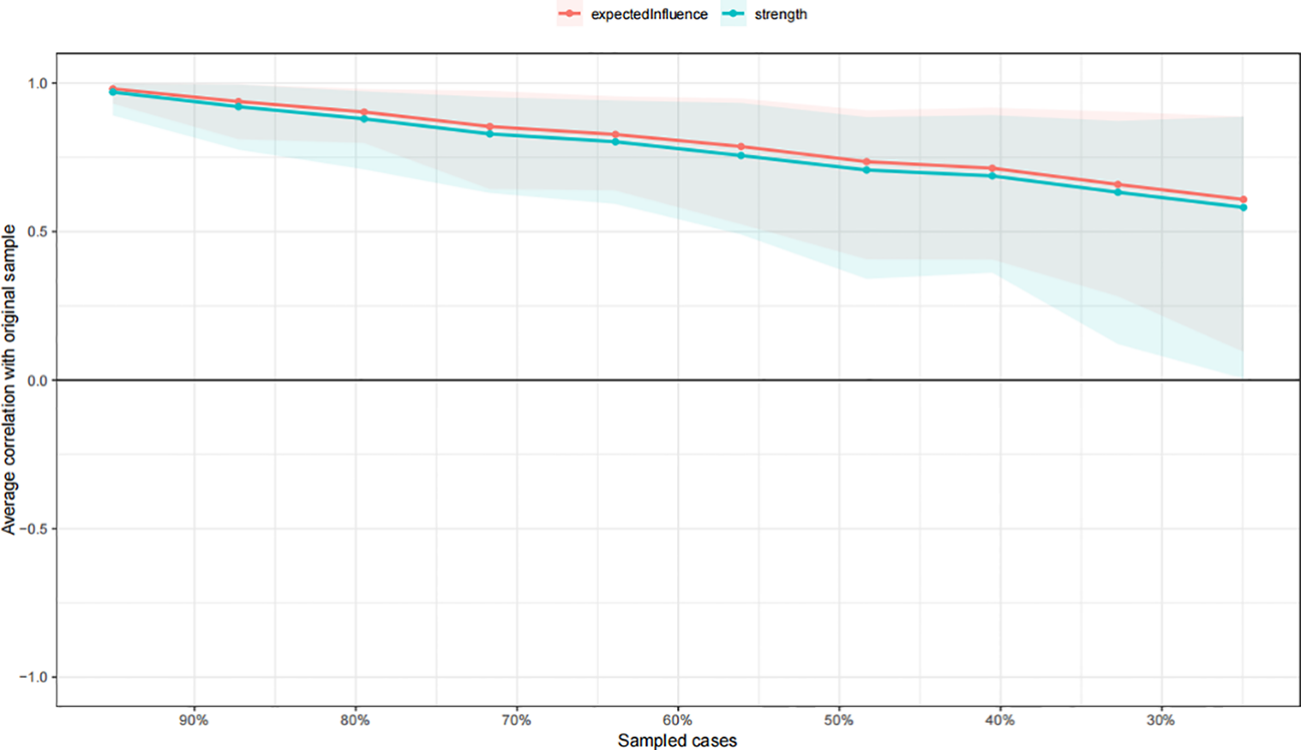
Stability of the symptom networks.

**Figure 3** presents the stability of the symptom networks based on bootstrapping analysis. With respect to network stability, the value of CS-C was 0.283 for the EI and strength, indicating that the network retained acceptable stability, as values above 0.25 are considered minimally sufficient. The results of the bootstrapped difference tests for edge weights and node centrality are shown in **Appendix S4, S5**, respectively. Regarding edge-weight differences, the edge connecting disinhibition, and the edge connecting apathy/indifference and night behavioral disturbances were significantly stronger than the majority of other edges. With respect to node-level difference tests, disinhibition and irritability/lability demonstrated significantly higher strength centrality than most other symptoms. The results of the bootstrap analysis (with 95% CIs) for edges are shown in **Appendix S6**.

## 4 Discussion

To our knowledge, this study is among the first to explore the NPS network in an independent sample of the older adults with MCI. By constructing an item-level NPI-Q symptom network, we characterized the contemporaneous associations among multiple NPS, providing a more refined understanding of how these symptoms cluster and may mutually reinforce one another in the MCI stage. Our findings showed that all 12 NPS nodes were interconnected. Based primarily on strength centrality and EI, disinhibition appeared to be the most influential symptom in the network, followed by irritability/lability and agitation/aggression. These results suggest that disinhibition, irritability/lability, and agitation/aggression may occupy relatively central positions within the NPS network of older adults with MCI and may be relevant to the overall organization of symptom burden. However, these findings should be interpreted with caution. Given that the stability of the network was only modest, the present results are better understood as exploratory. Accordingly, these potentially central symptoms may provide useful directions for future research and may warrant closer monitoring and more detailed assessment in future studies on NPS management in older adults with MCI.

### 4.1 Disinhibition was the most central symptom of NPSs among older adults with MCI

Our network analysis suggested that disinhibition may be the most central symptom in the NPS network, as indicated by its relatively high strength and EI and its direct connections with several other symptoms, including agitation/aggression, irritability/lability, anxiety, aberrant motor activity, and night-time behavioral disturbances. This configuration indicates that disinhibition may serve as a key marker of broader affective and behavioral dysregulation, closely linked to the overall NPS burden in older adults with MCI and, consequently, to increased caregiver burden. Our findings are partially consistent with previous work. Goodwin et al. also identified disinhibition and agitation/aggression as central symptoms (27), but their study was based on a mixed MCI and AD sample, whereas the present study focused specifically on older adults with MCI. This difference in sample composition may partly contribute to the observed variation in centrality patterns. In particular, irritability/lability emerged as an additional relatively central symptom in our network, which may reflect differences in symptom expression at earlier stages of cognitive impairment. Similarly, in a network study focusing on impulse dyscontrol symptoms in individuals with MCI and subjective cognitive decline, Saari et al. reported that stubbornness/rigidity, agitation/aggressiveness and argumentativeness formed a tightly interconnected core cluster (44). Although the symptom operationalization differs from that used in the present study, these findings also highlight the prominence of behavioral and impulse dyscontrol-related symptoms in earlier stages of cognitive impairment. Factor-analytic work on the NPI further supports a “hyperactivity/behavioral problems” factor, comprising agitation/aggression, disinhibition, irritability/lability and aberrant motor behavior, which closely mirrors the cluster of neighbors around disinhibition in our network (45).

Disinhibition and agitation/aggression are the most interconnected nodes in the network, which is consistent with prior work showing that these two symptoms often co-occur and cluster within a shared “hyperactivity/impulse-dyscontrol” domain (27, 45). Disinhibition refers to difficulty suppressing inappropriate or maladaptive thoughts or behaviors (46). Agitation is defined as inappropriate verbal, vocal, or motor activity which is not explained by apparent needs or confusion per se, which includes behavior such as aimless wandering, pacing, cursing, screaming, biting, and fighting (47). Aggression refers to more marked verbal insults (e.g., shouting, cursing) and physical behaviors (e.g., hitting, kicking, biting, throwing objects (27). Neurobiologically, both symptoms have been related to dysfunction of frontal-subcortical circuits, especially orbitofrontal and ventromedial prefrontal regions and their connections with limbic structures, which are crucial for behavioral inhibition, impulse control and regulation of anger (48, 49). Neuroimaging studies in AD and MCI have further shown that greater agitation and aggression severity is associated with atrophy or hypometabolism in frontolimbic regions, supporting a partly shared neuroanatomical substrate for disinhibited and agitated behavior (49, 50). Second, irritability is conceptualized as an affective manifestation of impaired emotional regulation, whereas disinhibition reflects behavioral inhibition failure. Clinically, this tight coupling indicates that emerging irritability/lability in older adults with MCI may signal early impairment in inhibitory control mechanisms, and interventions targeting disinhibition may produce downstream reductions in irritability and affective instability. Third, heightened anxiety may increase physiological arousal and threat vigilance (51), while concomitant frontal–executive dysfunction reduces the capacity to inhibit impulsive responses (52). This combination may create a vulnerability state in which anxious distress is preferentially channeled into disinhibited or risky behaviors, rather than remaining confined to internally experienced symptoms. Fourth, aberrant motor behaviors, as typically defined in dementia, include pacing, excessive fidgeting, repetitive handling or picking of objects, and aimless wandering. When behavioral inhibition is compromised, internally driven urges and restlessness are less likely to be suppressed or channeled into goal-directed activity, increasing the likelihood of purposeless or stereotyped motor acts. Finally, night-time behavioral disturbances in dementia and MCI typically manifest as difficulties initiating or maintaining sleep, fragmentation of the sleep, increased nocturnal awakenings, excessive daytime sleepiness, and, in some cases, nocturnal wandering or engaging in inappropriate activities at night. Network analyses in mixed MCI and AD samples have similarly shown that disinhibition was linked to aberrant motor activity, aberrant motor disturbance was associated with hallucinations, hallucinations were associated with nighttime behaviors, suggesting a cascading pathway from impulse dyscontrol to nocturnal disruption within the broader NPS network (27).

Given its central position in the network, disinhibition may represent a particularly promising intervention target for mitigating the overall NPS burden in older adults with MCI. Existing evidence suggests that disinhibition can be improved through pharmacological management as well as nonpharmacological interventions (e.g., models of care, education/training, physical activity, and music). Notably, the quality of research in RCTs was strong with a greater effect size in nonpharmacological compared to pharmacological approaches (53). From a network perspective, prioritizing interventions that target a highly central symptom is theoretically advantageous, as improvements in this node may propagate to multiple interconnected symptoms. Accordingly, nurses and other healthcare professionals may consider placing greater emphasis on the early identification and management of disinhibition when developing care plans for older adults with MCI. Rather than addressing NPS in isolation, such an approach encourages proactive, upstream intervention aimed at disrupting symptom reinforcement pathways, potentially reducing overall symptom complexity and caregiver burden. Importantly, embedding non-pharmacological strategies into routine care aligns with current best-practice recommendations for NPS management in the early stages of cognitive impairment.

### 4.2 The role of irritability/lability as a secondary central symptom of NPSs among older adults with MCI

In addition to disinhibition, irritability/lability emerged as a highly influential node in the NPS network of older adults with MCI, ranking second in both strength centrality and EI. While irritability/lability was also highly prevalent in this population, its central role in the network cannot be attributed solely to symptom frequency. Rather, its strong and widespread connections with multiple NPSs indicate that irritability/lability occupies a structurally influential position, suggesting a key role in the co-occurrence and mutual reinforcement of affective and behavioral symptoms during the MCI stage.

The prominent network position of irritability/lability in MCI may be understood in light of early disruptions in emotional regulation and executive control. Irritability is characterized by heightened emotional reactivity, low frustration tolerance, and impaired modulation of affective responses, processes that rely heavily on prefrontal-limbic circuitry (54). In individuals with MCI, subtle deficits in executive function and inhibitory control are common, even when global cognition remains relatively preserved (55, 56). These early regulatory impairments may predispose individuals to emotional lability, rendering irritability an early and sensitive manifestation of affective dyscontrol. As a result, irritability may emerge as a key symptom through which cognitive vulnerability translates into broader neuropsychiatric disturbance.

Importantly, irritability/lability appears to form direct connections with several other nodes, including agitation/aggression, disinhibition, appetite/eating abnormalities, anxiety, and apathy/indifference. Previous studies have shown that irritability has been consistently associated with both internalizing symptoms, such as anxiety (57), and externalizing symptoms, including agitation, aggression, and disinhibition (27). Notably, irritability and apathy/indifference may reflect not entirely independent processes. While apathy primarily involves reduced motivation and emotional responsiveness, irritability can exacerbate apathy by increasing frustration and emotional strain, particularly when cognitive and motivational resources are limited.

### 4.3 Agitation/aggression as a central driver of NPS symptom network of older adults with MCI

Agitation/aggression emerged as another highly central node in the NPS network of older adults with MCI, ranking immediately after disinhibition and irritability/lability. Its prominent network position suggests that agitation/aggression is not merely a behavioral manifestation, but rather a key driver symptom that may actively contribute to the propagation and amplification of neuropsychiatric disturbances during the MCI stage. The finding is consistent with the network analysis reported by Goodwin et al. in samples including individuals with MCI and Alzheimer’s disease (27).

In the network, agitation/aggression showed direct connections with disinhibition irritability/lability and depression/dysphoria. This suggests that agitated and aggressive behaviors may reflect the behavioral expression of underlying emotional distress and impaired regulatory control. Disinhibition refers to difficulty suppressing inappropriate or maladaptive thoughts or behaviors (46), while irritability/lability is commonly conceptualized as reflecting heightened emotional reactivity together with deficits in affect regulation (57). When both inhibitory control and emotional regulation are compromised, internal affective distress or environmental stressors may be more likely to manifest as overt agitation or aggressive behavior rather than remaining confined to internal emotional experiences.

Agitation/aggression is among the most burdensome NPS for caregivers and healthcare systems, often prompting increased healthcare utilization, psychotropic medication use, and consideration of institutional care (58, 59). Its central position in the symptom network suggests that agitation/aggression may play an important role in sustaining overall neuropsychiatric distress, even if it is not the primary cause of other symptoms. Therefore, early identification and management of agitation/aggression, preferably through non-pharmacological, person-centered approaches, may help reduce symptom escalation and caregiver burden.

### 4.4 Limitations

Several limitations of this study should be acknowledged. First, this study adopted a cross-sectional design and convenience sampling, which restricts causal inference and limits the generalizability of the findings. In particular, cross-sectional symptom networks capture contemporaneous conditional associations and cannot determine temporal ordering or directional pathways among symptoms; therefore, the dynamic evolution of NPS and their potential feedback processes over time remain uncertain. Future longitudinal studies are needed to construct temporal or dynamic networks and to examine whether central symptoms prospectively predict changes in other NPS. Second, participants were recruited from community and nursing-home settings, and individuals in hospital-based contexts were excluded, which may have reduced representation of more severe or clinically complex cases. As symptom profiles and care contexts can differ substantially across living environments, subsequent studies should validate the network structure in diverse settings (e.g., outpatient clinics, inpatient wards, and other long-term care models) and in populations with broader vulnerability profiles. Third, the sample was drawn from older adults residing in northern China, which may further constrain external validity. Cultural norms, social support structures, and regional health conditions may influence both symptom reporting and symptom co-occurrence patterns; thus, replication across different regions and countries is warranted to strengthen the generalizability and applicability of the present network findings. Fourth, measurement-related limitations should be considered. The item-level network was estimated using binary symptom presence/absence, which may obscure clinically meaningful variation in symptom severity and frequency, and may attenuate sensitivity to change. Future research could incorporate severity ratings or multi-informant assessments to improve measurement precision. Fifth, although a range of sociodemographic and clinical variables were collected and described, these variables were not incorporated as covariates in the network estimation. As a result, potential confounding effects of factors such as age, BMI, religious belief, location, employment status, living conditions, monthly income, smoking, drinking alcohol, and self-reported health status on symptom associations cannot be ruled out. Future studies may consider integrating relevant covariates to further examine the robustness of the network structure across different individual characteristics. Finally, MCI in this study was identified using an operational definition based on screening measures, including subjective cognitive complaint, MoCA performance, preserved basic functional independence on the ADL scale, and absence of a prior clinical diagnosis of dementia, rather than formal clinical diagnosis according to established criteria. This approach may introduce potential misclassification between normal aging, MCI, and early dementia, which could influence the interpretation of the findings. Future studies should adopt standardized clinical diagnostic criteria and incorporate more comprehensive clinical assessments to improve the accuracy of MCI classification.

## 5 Conclusion

This study employed a network analysis to examine the structure of NPS among older adults with MCI. The results showed that disinhibition, irritability/lability, and agitation/aggression were the most central symptoms in the network. The findings suggested that disinhibition, irritability/lability, and agitation/aggression occupied relatively central positions in the network, indicating that greater attention to these potentially central symptoms may provide a useful basis for future research on stratified assessment and targeted intervention strategies for NPS in older adults with MCI.

## 6 Implication

The identification of disinhibition, irritability/lability, and agitation/aggression as potentially central symptoms in the NPS network suggests that symptom assessment in older adults with MCI may benefit from a network-informed perspective. Rather than managing symptoms solely based on their prevalence, focusing on symptoms with greater structural influence may help identify key drivers of broader neuropsychiatric disturbance, thereby improving the efficiency of screening and the rational allocation of healthcare resources. From a nursing and clinical research perspective, these findings may support closer monitoring and more detailed assessment of disinhibition, irritability/lability, and agitation/aggression in future studies of NPS in older adults with MCI. However, the present findings should be interpreted with caution. Given that the network stability coefficient reached only the minimally acceptable threshold, and that the results were derived from a cross-sectional sample drawn primarily from community and nursing-home settings in northern China, the centrality findings are better understood as exploratory rather than directly practice-guiding. Future studies should replicate these results in larger and more diverse samples and use longitudinal designs to determine whether changes in these symptoms are associated with broader changes in network structure over time.

## Data Availability

The raw data supporting the conclusions of this article will be made available by the authors, without undue reservation.
