# Supplementary material for: Identification of the central symptoms of neuropsychiatric symptoms among older adults with mild cognitive impairment: a network analysis
Source: Front Psychiatry. 2026 Apr 29;17:1820870. doi: 10.3389/fpsyt.2026.1820870 (PMC13168141; doi:10.3389/fpsyt.2026.1820870)
Supplement: Supplementary file 1 [file Supplementaryfile1.docx]

**Supplementary material**

**Contens**

[Appendix S1. Sociodemographic and Clinical Characteristics Questionnaire 2](#_Toc225239406)

[Appendix S2. The flowchart of the recruitment process. 4](#_Toc225239407)

[Appendix S3. The centrality indices of symptom network 5](#_Toc225239408)

[Appendix S4. Bootstrapped difference test for edges 6](#_Toc225239409)

[Appendix S5. Bootstrapped difference test for nodes. 7](#_Toc225239410)

[Appendix S6. Accuracy of the symptom networks 8](#_Toc225239411)

# Appendix S1. Sociodemographic and Clinical Characteristics Questionnaire

1. Age (years): ___
2. Gender: □ Male □ Female
3. Body Mass Index (BMI, kg/m²): Height: _____ cm, Weight: _____ kg
4. Ethnicity: □ Han Chinese □ Ethnic minority
5. Religious belief: □ Yes □ No
6. Education level: □ Illiterate □ Primary elementary □ Junior high school □ Senior high school □ Graduate or above
7. Location: □ Urban □ Non-urban
8. Marital status: □ Unmarried □ Married
9. Employment status: □ Employed □ Retired □ Unemployed □ Others
10. Living conditions: □ Live alone □ Live with parents □ Live with children □ Live with spouse □ Live with other friends
11. Monthly income: □ ￥2000 or less □ ￥2001-￥5000 □ ￥5001-￥10000 □ ￥10001 or more
12. Smoking: □ Yes □ No
13. Drinking alcohol: □ Yes □ No
14. Self-reported health status: □ Healthy □ Not healthy, not unhealthy □ Unhealthy
15. Social support: □ Yes □ No
16. Parental history of dementia: □ Yes □ No
17. Chronic diseases

| **Condition** | **Yes/No** | **Condition** | **Yes/No** |
| --- | --- | --- | --- |
| History of stroke |  | History of long-term malnutrition |  |
| History of epilepsy |  | Hypothyroidism |  |
| Brain tumor |  | History of heavy use of sedative or analgesic medications |  |
| History of head trauma |  | History of gas poisoning or chronic alcohol intoxication |  |
| History of encephalitis |  | Diabetes mellitus |  |
| History of Parkinson’s disease |  | Hypertension |  |
| Heart failure |  | Hyperlipidemia |  |
| Coronary heart disease |  | Emotional disorders (e.g., anxiety, depression) |  |
| Midlife obesity |  | Recent weight loss |  |
| History of surgery |  | History of major life events (e.g., death of a loved one) |  |
| Cognitive impairment (Alzheimer’s disease) |  | Sleep disorders (e.g., insomnia) |  |

# Appendix S2. The flowchart of the recruitment process.

**
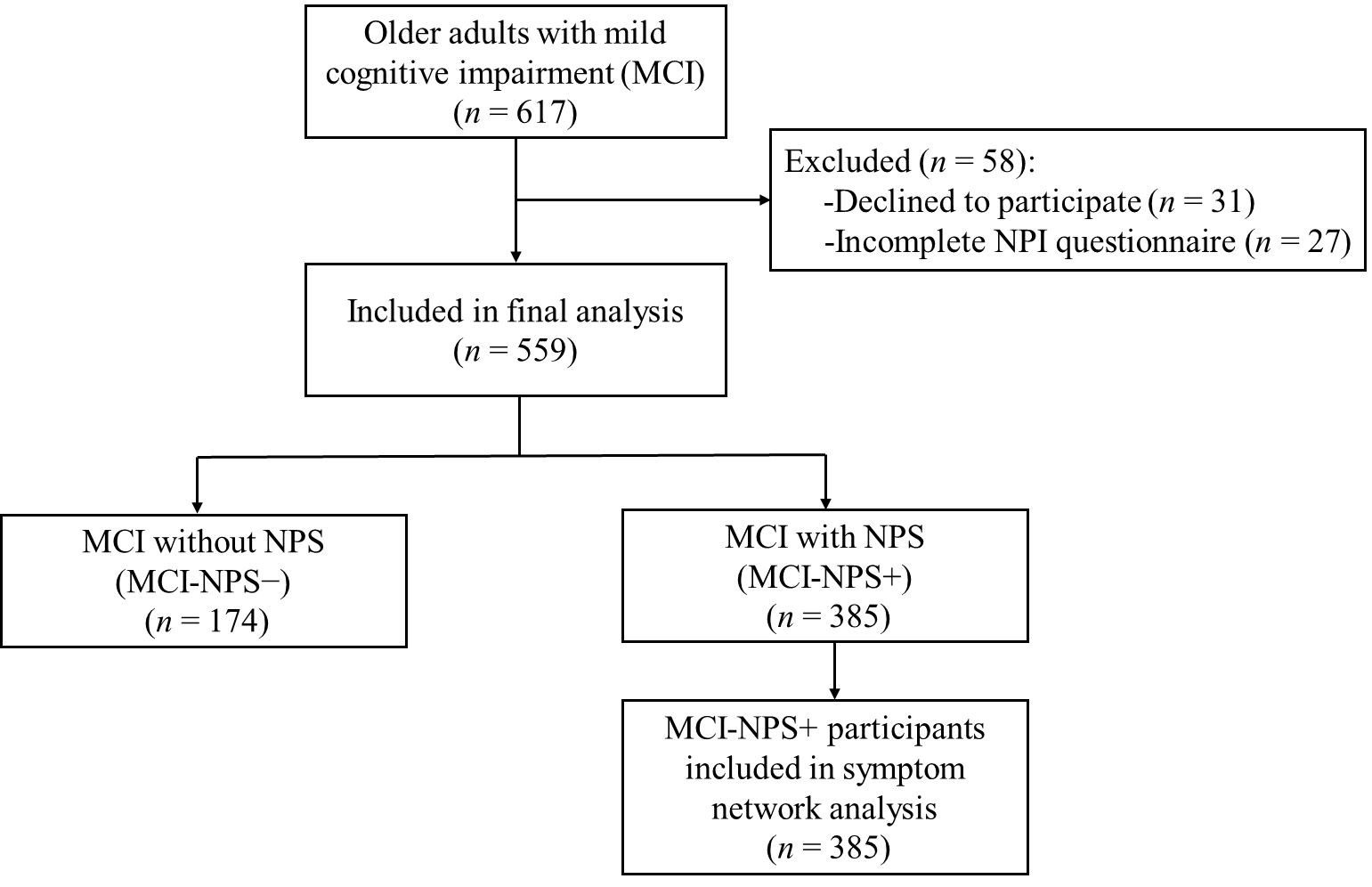
**

# Appendix S3. The centrality indices of symptom network

| **Item** | **Betweenness** | **Closeness** | **Strength** | **Expected Influence** |
| --- | --- | --- | --- | --- |
| NPI-1 | 0 | 0.026122 | 2.349350 | 2.349350 |
| NPI-2 | 10 | 0.029390 | 3.179584 | 3.179584 |
| NPI-3 | 15 | 0.052336 | 5.829463 | 5.829463 |
| NPI-4 | 18 | 0.041002 | 2.560604 | 2.560604 |
| NPI-5 | 3 | 0.037421 | 1.978791 | 1.978791 |
| NPI-6 | 0 | 0.021216 | 0.973372 | 0.973372 |
| NPI-7 | 0 | 0.032849 | 2.171307 | 2.171307 |
| NPI-8 | 30 | 0.051971 | 6.822706 | 6.822706 |
| NPI-9 | 13 | 0.048961 | 6.065493 | 6.065493 |
| NPI-10 | 10 | 0.027129 | 1.427415 | 1.427415 |
| NPI-11 | 0 | 0.032699 | 1.670885 | 1.670885 |
| NPI-12 | 0 | 0.035925 | 1.707984 | 1.707984 |

# Appendix S4. Bootstrapped difference test for edges


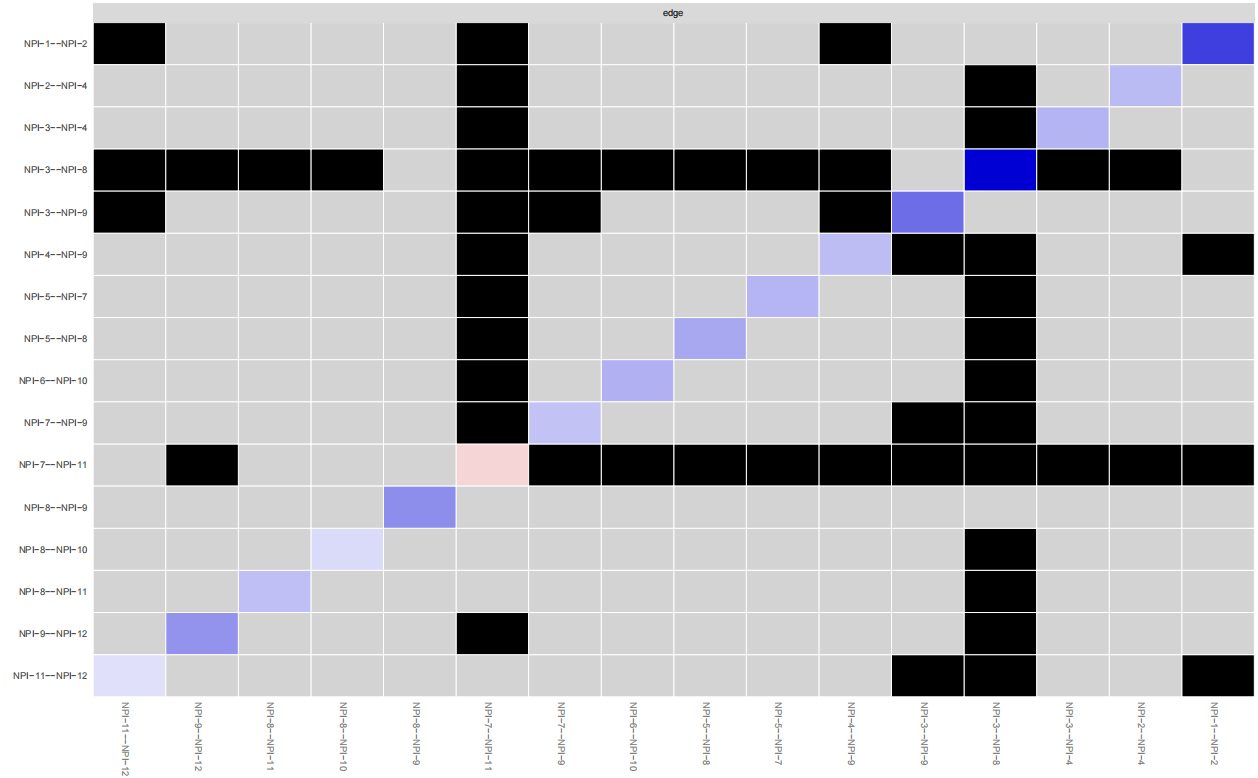


Appendix 2. Bootstrapped difference test for edges

# Appendix S5. Bootstrapped difference test for nodes.


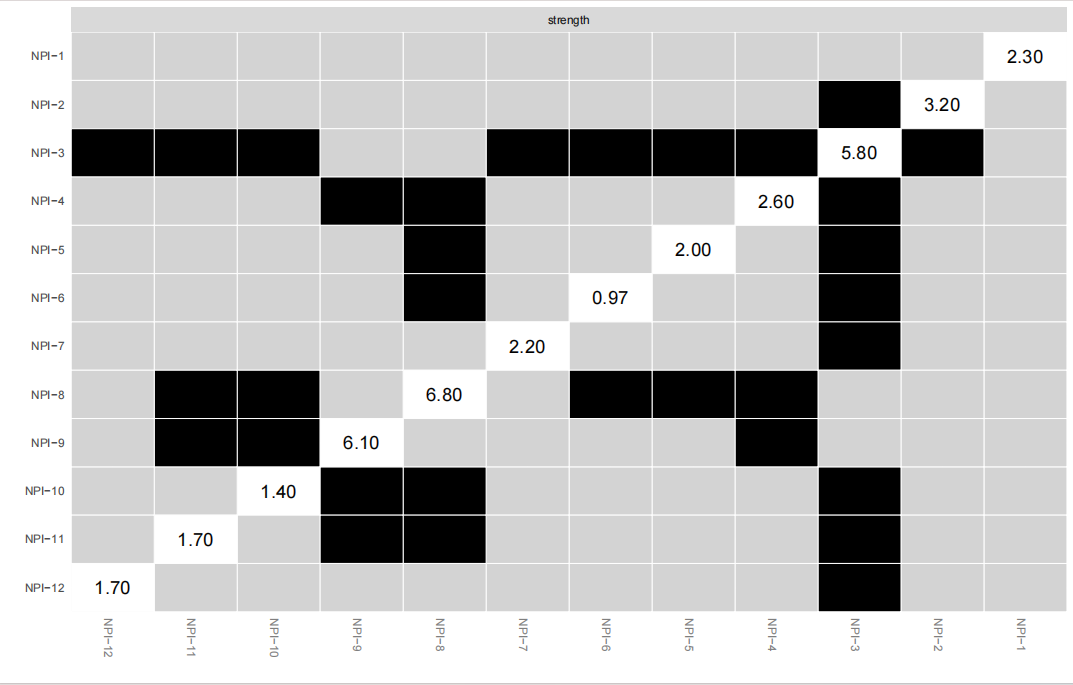


Appendix 3. Bootstrapped difference test for nodes.

# Appendix S6. Accuracy of the symptom networks


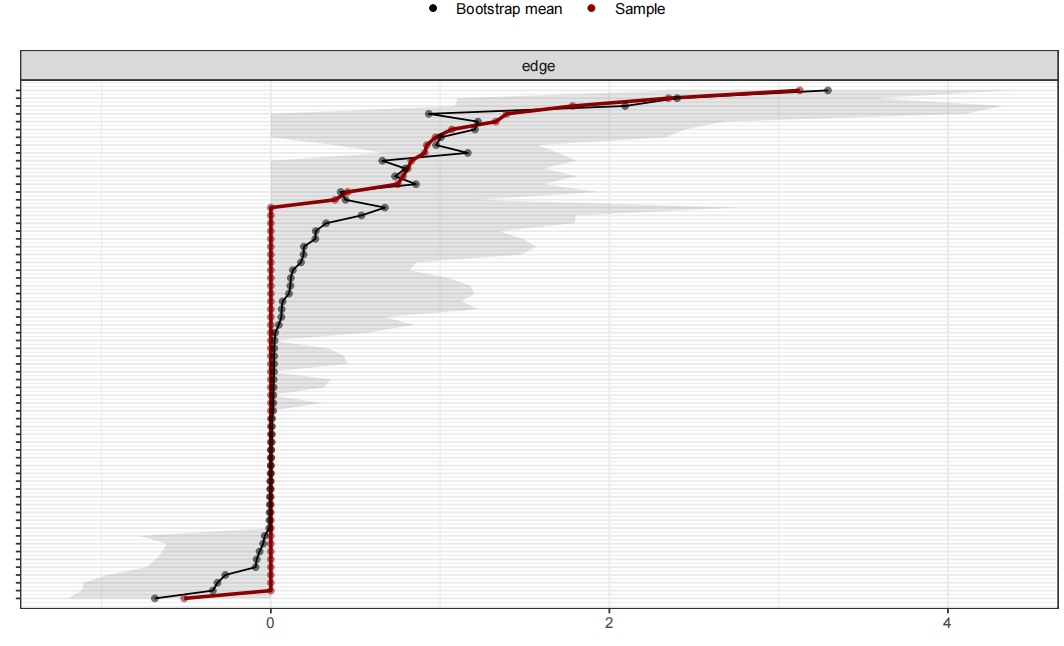


Appendix 4. Accuracy of the symptom networks
